# Intra-seasonal asymmetry in satellite-observed vegetation greenness changes and their climatic associations in arid Northwest China

**DOI:** 10.3389/fpls.2026.1897977

**Published:** 2026-08-13

**Authors:** Xigang Liu, Ziyi Zhuang, Xiqian Wei, Zhengyi Xi

**Affiliations:** Xinjiang Laboratory of Lake Environment and Resources in Arid Zone, College of Geographic Science and Tourism, Xinjiang Normal University, Urumqi, China

**Keywords:** climatic associations, arid ecosystems, intra-seasonal asymmetry, month-to-month NDVI changes, vegetation greenness

## Abstract

Arid ecosystems are highly sensitive to climate change, yet it remains unclear whether regional greening is accompanied by consistent changes in vegetation greenness throughout the growing season. Using SPOT/VEGETATION NDVI and meteorological data from 1999 to 2018, we applied a month-to-month NDVI difference metric (V_NDVI_) to characterize intra-seasonal variation in satellite-observed vegetation greenness and to examine its relationships with concurrent and antecedent climatic conditions across arid Northwest China. May–October was defined as the common analytical window for regional comparison, with May–July representing the early-to-peak growing season and August–October representing the late growing season. Although NDVI exhibited positive long-term trends in most months, VNDVI trends showed pronounced intra-seasonal asymmetry. VNDVI increased significantly in June and July but decreased significantly in October, indicating that regional greening was accompanied by a redistribution of month-to-month vegetation greenness changes rather than a uniform enhancement throughout the growing season. These trends also exhibited marked spatial heterogeneity. Negative V_NDVI_ trends in May were concentrated in the Altai Mountains and the western Tianshan Mountains, whereas positive trends in June and July were most evident in the central Tianshan Mountains and along the northern margin of the Tarim Basin. The relationships between V_NDVI_ and climatic variables varied among months and regions. Precipitation was generally positively associated with month-to-month vegetation greenness changes, whereas temperature and radiation showed negative associations in several months and regions. Hierarchical partitioning further revealed that the relative independent contributions of climatic variables to NDVI variability differed among vegetation types, with temperature accounting for a particularly large proportion of the explained variation in croplands during the early growing season.

## 1 Introduction

Arid and semi-arid ecosystems are particularly sensitive to climate variability and long-term environmental change because vegetation activity is strongly constrained by limited and highly variable water availability (Kong et al., 2026). These regions occupy a substantial proportion of the terrestrial land surface and support large human populations (Li et al., 2021; Gong et al., 2024), while also providing important ecosystem functions, including carbon uptake, soil stabilization, and regulation of regional water and energy exchanges. Understanding how vegetation greenness responds to changing climatic conditions is therefore important for assessing the vulnerability and resilience of dryland ecosystems (Kong et al., 2026; Ma et al., 2026).

Satellite-derived vegetation indices, particularly the normalized difference vegetation index (NDVI), have been widely used to document long-term greening across arid and semi-arid regions (Jia et al., 2025; Cui et al., 2026; Zhang et al., 2026). Most previous studies have focused on annual or growing-season mean NDVI, growing-season integrals, or phenological transition dates. These metrics provide important information on broad changes in vegetation greenness but may obscure substantial variation within the growing season (Li et al., 2023; Qi et al., 2025). In particular, an increase in seasonal mean NDVI does not necessarily imply that vegetation greenness has increased uniformly across all months. Regional greening may instead reflect stronger increases during particular parts of the season, shifts in the timing of peak greenness, or changes in the magnitude of NDVI differences between consecutive months.

Month-to-month NDVI differences provide a simple means of characterizing changes in the seasonal NDVI trajectory. Following the framework of Piao et al. (2022), V_NDVI_ is defined as the difference between NDVI in a target month and that in the immediately preceding month. Unlike absolute NDVI, which represents the level of satellite-observed surface greenness, V_NDVI_ describes the direction and magnitude of change in NDVI between consecutive months. Positive and negative V_NDVI_ values therefore indicate monthly increases and decreases in NDVI, respectively, rather than direct changes in biomass accumulation, canopy structure, or physiological growth. Examining long-term trends in V_NDVI_ can reveal whether the seasonal distribution of month-to-month greenness changes has shifted over time.

Climatic conditions may be associated with both absolute vegetation greenness and month-to-month changes in greenness, but these associations can differ among months and vegetation types. In water-limited environments, precipitation is often positively associated with vegetation activity, whereas the relationships with temperature and solar radiation may vary with seasonal water availability and atmospheric demand (Jiao et al., 2021; Mao et al., 2026). Moreover, vegetation responses may be associated not only with climate in the target month but also with conditions in preceding months. Such antecedent associations may reflect delayed ecosystem responses or temporal covariance among climatic and vegetation variables, but they should not be interpreted automatically as evidence of specific physiological or ecohydrological mechanisms. Distinguishing associations with NDVI from those with V_NDVI_ is therefore necessary because climatic conditions related to vegetation greenness levels may differ from those related to changes between consecutive months.

The arid region of Northwest China is characterized by strong climatic gradients (Zhu et al., 2024), complex topography, and marked spatial heterogeneity in vegetation cover (Lu et al., 2025). Vegetated areas are concentrated in mountain systems, oasis margins, and desert–oasis ecotones, where vegetation is exposed to substantial variation in temperature, precipitation, and radiation. Previous studies have reported widespread greening in this region, but most have emphasized annual or growing-season changes (Jia et al., 2024; Zhang et al., 2024; Fu et al., 2026). It remains unclear whether this regional greening has been accompanied by consistent changes in month-to-month vegetation greenness throughout the growing season, whether such changes differ spatially and among vegetation types, and how they are statistically associated with concurrent and antecedent climatic conditions.

Here, we combined SPOT/VEGETATION NDVI data and gridded meteorological records for 1999–2018 to examine intra-seasonal variation in vegetation greenness across arid Northwest China. We used V_NDVI_ to characterize month-to-month NDVI changes, quantified long-term temporal and spatial trends in NDVI and V_NDVI_, and assessed their conditional associations with concurrent and antecedent temperature, precipitation, and radiation. We further used hierarchical partitioning to compare the relative independent explanatory contributions of these climatic variables to interannual NDVI variability across vegetation types. Specifically, we addressed three questions: (1) Do month-to-month NDVI changes exhibit temporal and spatial asymmetry within the common May–October analytical window? (2) Which months contribute most strongly to long-term changes in the seasonal NDVI trajectory? and (3) How are monthly NDVI and V_NDVI_ associated with concurrent and antecedent climatic conditions, and how do the relative explanatory contributions to NDVI variability differ among vegetation types?

## 2 Materials and methods

### 2.1 Study area

The arid region of Northwest China (ARNC; 73°–123° E, 32°–50° N) is located in the interior of the Eurasian continent and is characterized by a temperate continental climate, limited precipitation, high evaporative demand, and strong spatial variability in hydroclimatic conditions (Yang et al., 2026). The region contains extensive deserts, mountain systems, oasis landscapes, and desert–oasis ecotones, resulting in pronounced spatial heterogeneity in vegetation cover and climatic conditions (Wang et al., 2022).

**Figure 1:**
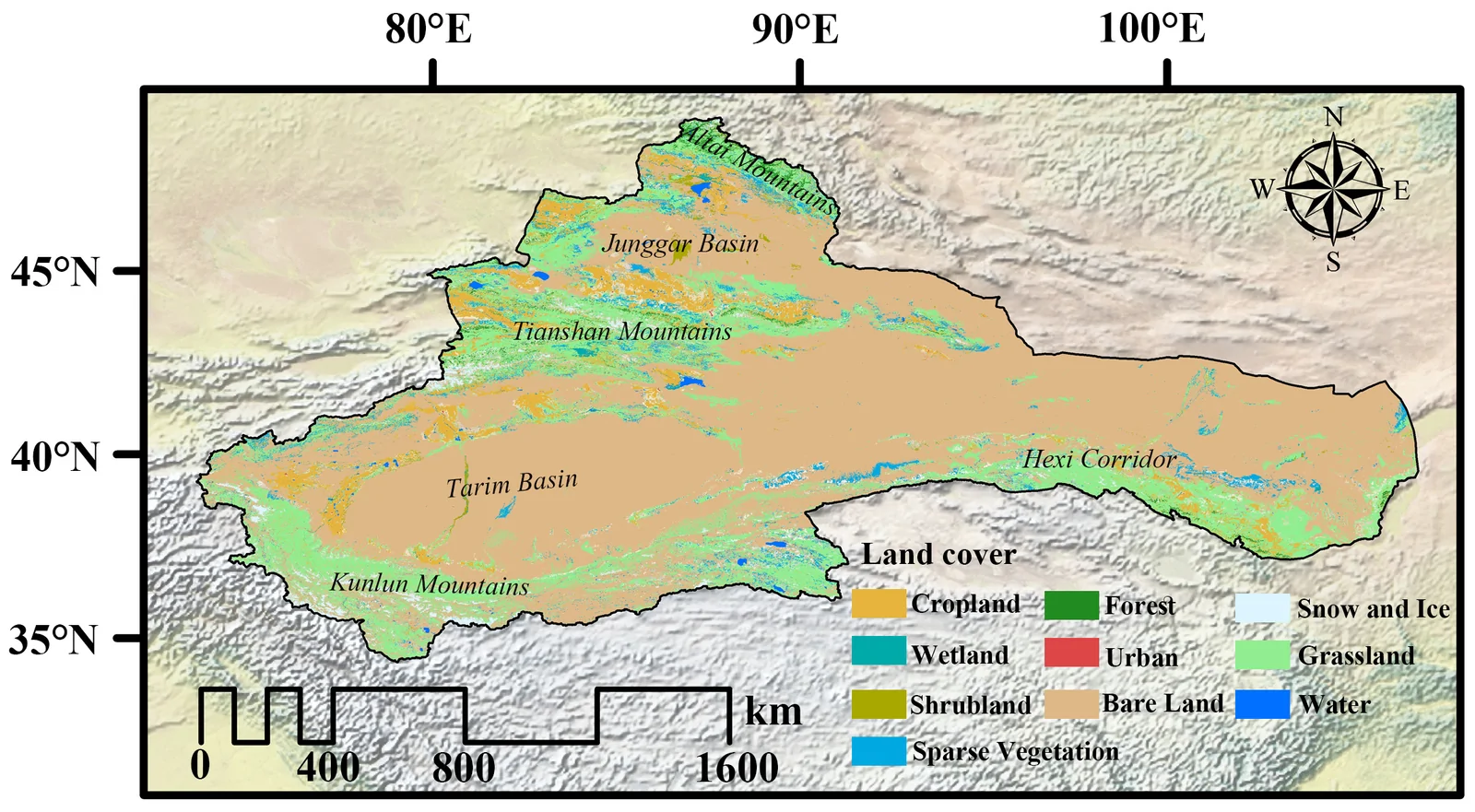
Location and land cover of the study area.

To examine differences among vegetation types, we used the annual land-cover product from the European Space Agency Climate Change Initiative (ESA CCI) (Hollmann et al., 2013). The dataset covers 1992–2018, has an original spatial resolution of 300 m, and contains 37 land-cover classes. These classes were reclassified into 10 broad categories: cropland, grassland, forest, urban areas, water bodies, shrubland, wetlands, bare land, sparse vegetation, and permanent snow and ice (**Figure 1**). To reduce the influence of land-cover conversion on the NDVI and V_NDVI_ analyses, we retained only pixels that remained consistently classified as cropland, grassland, shrubland, forest, or sparse vegetation throughout 1992–2018. The reclassified land-cover maps were resampled to the 1-km NDVI grid using nearest-neighbor resampling to preserve categorical class values.

### 2.2 NDVI dataset

Monthly normalized difference vegetation index (NDVI) data were obtained from the 1-km monthly vegetation index dataset derived from the SPOT/VEGETATION satellite observations. This dataset provides long-term satellite-based observations of surface vegetation greenness at a spatial resolution of 1 km and has been widely used for regional-scale vegetation monitoring and ecological analyses. The original SPOT/VEGETATION observations were acquired at 10-day intervals and aggregated into monthly composites using the maximum-value composite (MVC) method to reduce the influence of cloud contamination and other observation uncertainties (Xu, 2018).

The available record covered April 1998 to June 2020. To ensure complete calendar years for temporal trend analysis and climate-association analyses, we used monthly NDVI data from January 1999 to December 2018. All analyses were conducted using the original 1-km spatial resolution.

Because NDVI primarily represents satellite-observed surface greenness rather than direct measurements of vegetation structure, biomass accumulation, or physiological growth, we interpreted NDVI variations as changes in vegetation greenness throughout the study. To reduce the influence of sparsely vegetated and non-vegetated surfaces, we calculated the mean May–October NDVI for each pixel over 1999–2018 and excluded pixels with values below 0.1. The resulting vegetation mask was consistently applied to all subsequent NDVI, V_NDVI_, climatic-association, and hierarchical-partitioning analyses.

### 2.3 Climate datasets

Monthly air temperature, precipitation, and surface solar radiation data were obtained from the CHELSA V2.1 dataset (https://www.chelsa-climate.org/datasets/chelsa_monthly). The data have a spatial resolution of approximately 1 km and provide gridded monthly climatic variables suitable for regional-scale analyses (Karger and Zimmermann, 2021). We extracted monthly data for 1999–2018 and matched them to the spatial extent, projection, and cell alignment of the NDVI dataset.

These variables were selected to characterize thermal, water-supply, and radiative conditions potentially associated with monthly NDVI and V_NDVI_ variability. Because the present study was based on statistical associations, the selected climatic variables were not interpreted as direct measurements of physiological processes or as a complete representation of the mechanisms underlying vegetation responses.

### 2.4 Spatial preprocessing and raster alignment

All spatial datasets were clipped to the ARNC boundary and processed on the 1-km NDVI grid. The 300-m ESA CCI land-cover data were resampled using nearest-neighbor resampling, whereas the CHELSA climate layers were matched to the NDVI data in projection, spatial extent, cell size, and cell alignment. The same vegetation mask and stable land-cover mask were then applied to the NDVI, V_NDVI_, and climate datasets to ensure that all pixel-wise analyses were conducted using spatially corresponding observations.

Where spatial reprojection was required, continuous climatic variables were resampled using nearest-neighbor resampling. No additional spatial smoothing was applied before the pixel-wise analyses.

### 2.5 Calculation and interpretation of V_NDVI_

Following Piao et al. (2022), V_NDVI_ was calculated as the difference between NDVI in a target month and that in the immediately preceding month as shown in Equation 1:

(1) $$V_{NDVI}\left(t\right)=NDVI\left(t\right)-NDVI\left(t-1\right)$$

where *t* denotes the target month, *NDVI* (*t*) is NDVI in the target month, and *NDVI* (*t*-1) is NDVI in the preceding month.

Because the temporal interval was fixed at one month, V_NDVI_ represents the month-to-month change in satellite-observed NDVI. A positive V_NDVI_ value indicates that NDVI was higher in the target month than in the preceding month, whereas a negative value indicates that NDVI was lower. V_NDVI_ was therefore used as a descriptive metric of change along the seasonal NDVI trajectory and not as a direct measure of physiological growth, biomass accumulation, canopy structural development, or vegetation senescence (Myers-Smith et al., 2020; Park et al., 2020).

The sign of mean V_NDVI_ was interpreted separately from the sign of its long-term trend. Mean V_NDVI_ describes the average direction of the NDVI difference between two consecutive months, whereas the V_NDVI_ trend describes how this monthly difference changed over 1999–2018. Accordingly, a negative V_NDVI_ trend may indicate either a weakening positive monthly increment or an increasingly negative monthly difference, depending on the sign of mean V_NDVI_.

### 2.6 Trend analysis

Long-term trends in monthly NDVI and V_NDVI_ were estimated using the Theil–Sen median slope estimator (Theil, 1992). This non-parametric estimator is relatively insensitive to outliers and does not require the residuals to follow a normal distribution. The statistical significance of each temporal trend was evaluated using the Mann–Kendall test (Mann, 1945).

Trend analyses were conducted for each month within the May–October analytical window over 1999–2018. Regional trends were estimated from the annual regional mean series, whereas spatial trends were calculated independently for each valid pixel. Unless otherwise stated, statistical significance was assessed at *p*<0.05.

For interpretation, the trend in target-month V_NDVI_ is mathematically equivalent to the difference between the NDVI trends of the target month and the preceding month. V_NDVI_ trends were therefore interpreted in relation to both the target-month and preceding-month NDVI trends.

### 2.7 Selection of concurrent and antecedent climatic months

For each target month from May to October, we considered climatic conditions in the target month and in the three preceding months. These four candidate periods were represented as lags 0–3: lag 0 corresponded to the target month, lag 1 to the immediately preceding month, and lags 2 and 3 to two and three months before the target month, respectively.

For each pixel, target month, vegetation indicator, and climatic variable, Pearson correlation coefficients were calculated between the 1999–2018 annual series of NDVI or V_NDVI_ and the corresponding climatic series at lags 0–3. The lag yielding the largest absolute Pearson correlation coefficient was selected separately for temperature, precipitation, and radiation. The selected climatic month was therefore allowed to vary among pixels, target months, vegetation indicators, and climatic variables rather than being prescribed uniformly across the study region.

The selected series was subsequently used in the partial-correlation analysis. This screening procedure identified the concurrent or antecedent month showing the strongest statistical association with the target-month vegetation indicator. It did not identify an optimal physiological preseason, demonstrate a climatic legacy mechanism, or establish causality.

### 2.8 Partial correlation analysis

Partial correlation analysis was used to quantify the conditional associations of temperature, precipitation, and radiation with monthly NDVI and V_NDVI_. For each target month, vegetation indicator, and pixel, the climatic series selected by the lag-screening procedure was used in the analysis.

When assessing the association with one climatic variable, the selected series of the other two climatic variables were statistically controlled. For example, the first-order partial correlation between variables x and y, controlling for variable z, was calculated using Equation 2:

(2) $$r_{xy\times z}=\frac{r_{xy}-r_{xz}r_{yz}}{\sqrt{\left(1-r_{xz}^{2}\right)\left(1-r_{yz}^{2}\right)}}$$

Where *r_xy_*, *r_xz_*, and *r_yz_* denote the corresponding pairwise Pearson correlation coefficients. Partial correlations controlling for two covariates were obtained by sequentially removing the variation shared with both covariates.

Statistical significance was evaluated using a two-sided test at *p*<0.05. The resulting coefficients were interpreted as conditional statistical associations after accounting for covariation among the three climatic variables. They were not interpreted as independent climatic effects, causal controls, or evidence of specific physiological mechanisms.

### 2.9 Relative importance assessment

Because temperature, precipitation, and radiation may covary, their relative contributions to NDVI variability cannot be reliably inferred from marginal correlations alone. We therefore applied hierarchical partitioning (HP) as a complementary variance-partitioning approach to quantify the relative independent explanatory contributions of climatic variables.

The analysis was implemented using the hier.part package in R (Walsh and Li, 2013). For each valid pixel, target month, and vegetation type, the annual NDVI series during 1999–2018 was used as the response variable, and the corresponding temperature, precipitation, and radiation series selected through the lag-screening procedure were used as predictors (Lai et al., 2022). Hierarchical partitioning evaluates all possible combinations of predictors and estimates the independent contribution of each variable by averaging its contribution across all candidate models.

The pixel-level independent contributions of temperature, precipitation, and radiation were expressed as percentages of the summed independent contributions of all predictors. The resulting pixel-wise contributions were subsequently summarized within each vegetation type (grassland, shrubland, cropland, forest, and sparse vegetation) to characterize differences in climatic associations among vegetation types.

**Figure 2:**
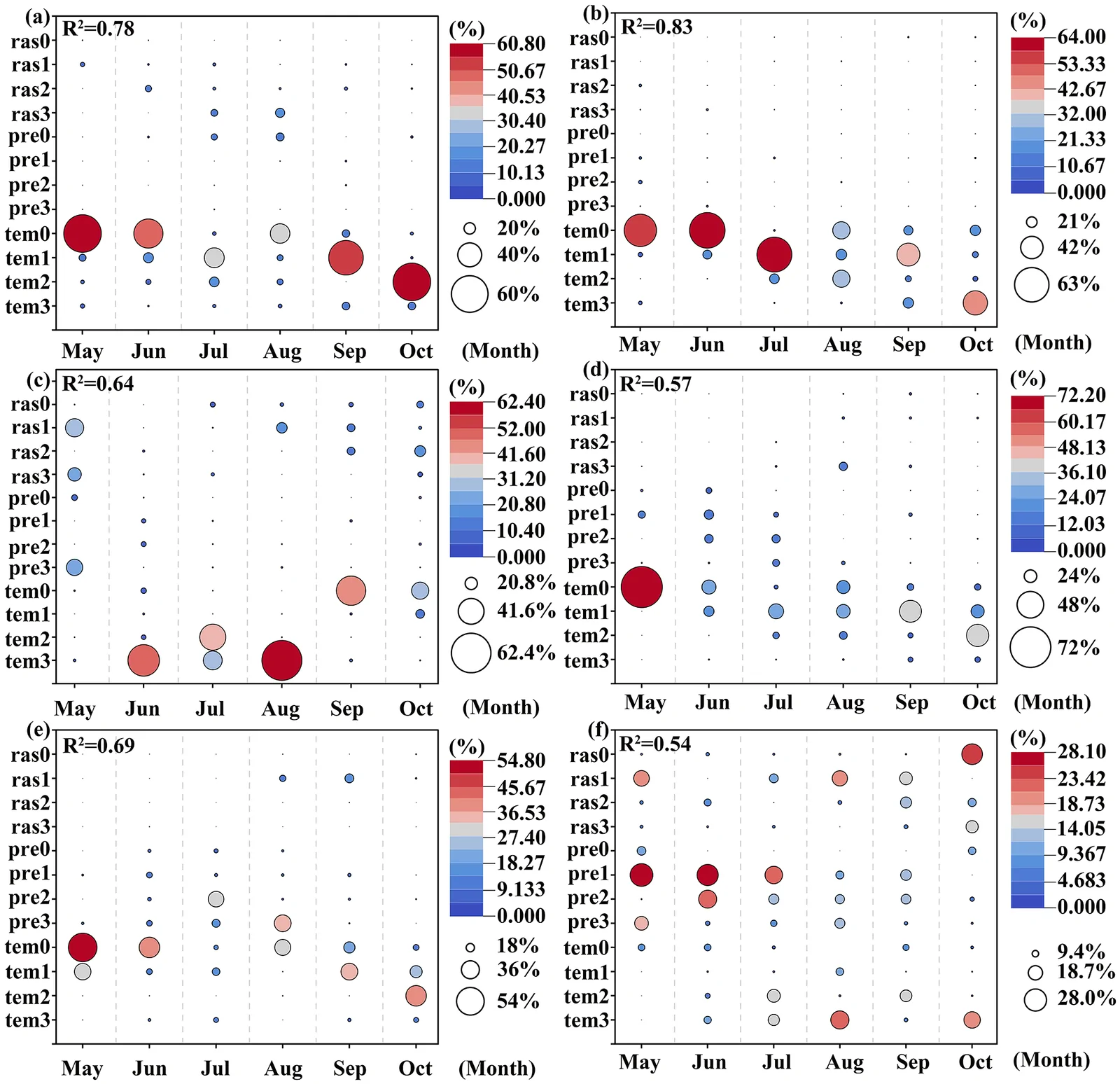
Relative independent explanatory contributions of selected concurrent and antecedent climatic variables to interannual NDVI variability across vegetation types. Panels **(a–f)** show hierarchical-partitioning results for **(a)** the entire study region, **(b)** grassland, **(c)** shrubland, **(d)** cropland, **(e)** forest, and **(f)** sparse vegetation. Hierarchical partitioning was conducted independently for each valid pixel, and the pixel-level results were summarized for the entire region and within each vegetation type. Bubble size and color represent the relative independent contribution of each climatic predictor, expressed as a percentage of the summed independent contributions of temperature, precipitation, and radiation. The R² value shown in each panel represents the mean pixel-level coefficient of determination explanatory capacity of the corresponding models.

These relative independent contributions represent the explanatory contribution of climatic variables within the fitted statistical models. The goodness-of-fit of each hierarchical partitioning model was evaluated using the explained variance (R²) of the corresponding regression model. The R² was calculated for each pixel-level model to evaluate the joint explanatory capacity of the three climatic predictors. Pixel-level R² values were subsequently summarized using the mean for the entire study region and for each vegetation type, and the resulting values are reported in **Figure 2**. They do not indicate causal effects, physiological responses, or ecological dominance of individual climatic factors (Xu et al., 2021; Kong et al., 2022).

## 3 Results

### 3.1 Spatiotemporal patterns and trends of V_NDVI_ in the ARNC

#### 3.1.1 Seasonal patterns and long-term changes in NDVI and V_NDVI_ in the ARNC

**Figure 3:**
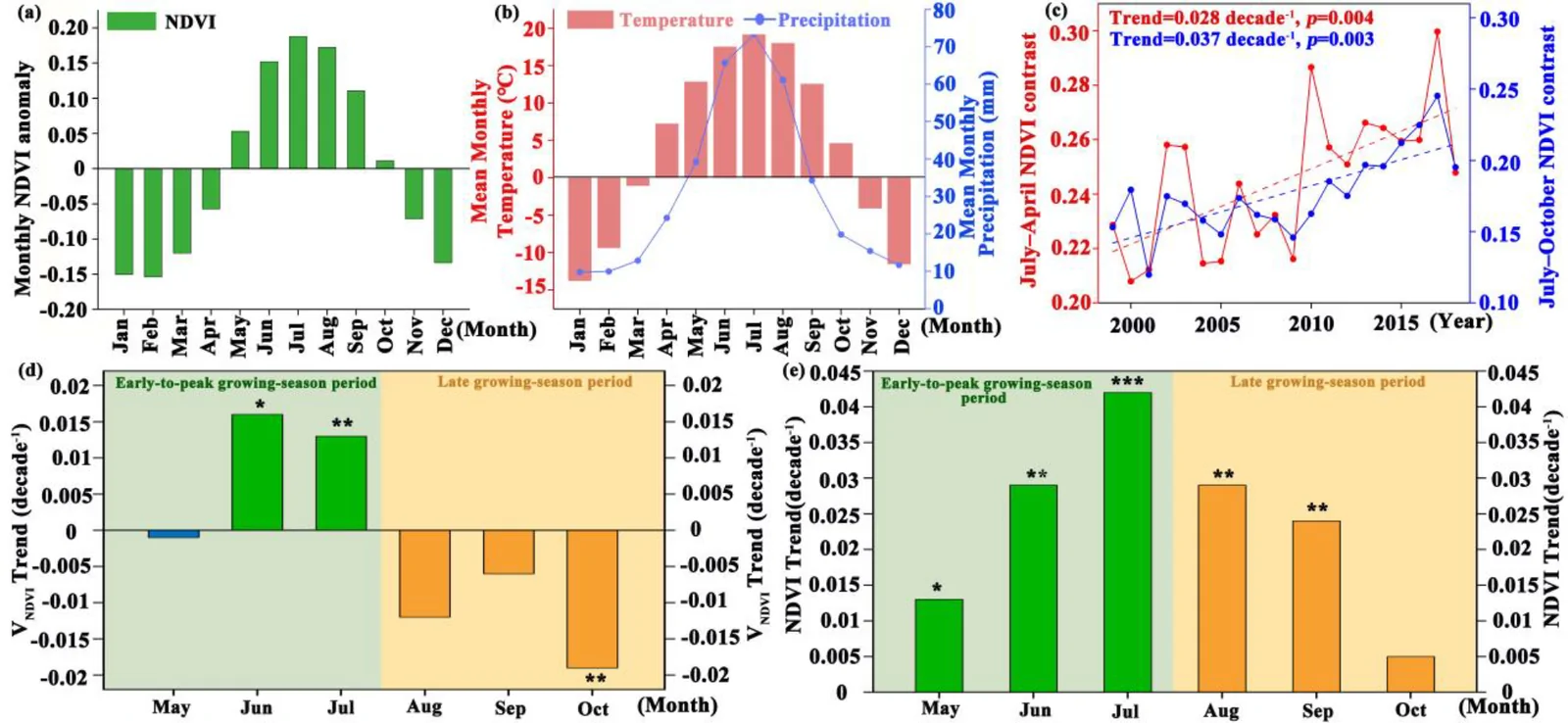
Seasonal patterns and long-term trends in NDVI and V_NDVI_ across the ARNC during 1999–2018. **(a)** Multi-year mean monthly NDVI anomalies, calculated as the difference between each monthly mean NDVI and the corresponding long-term annual mean NDVI. **(b)** Multi-year mean monthly temperature and precipitation. **(c)** Interannual variations and long-term trends in July–April and July–October NDVI contrasts. **(d)** Long-term trends in monthly V_NDVI_ during the May–October analytical window. **(e)** Long-term trends in monthly NDVI during the same period. Trend values in **(c–e)** are expressed per decade. Green and orange backgrounds denote the early-to-peak and late growing-season analytical periods, respectively. Asterisks indicate significance levels of p < 0.05, p < 0.01, and p < 0.001. V_NDVI_ is calculated as the target-month NDVI minus the NDVI of the preceding month.

The multi-year monthly NDVI climatology showed a clear seasonal pattern across the ARNC during 1999–2018. Mean NDVI increased from May (0.257) to June (0.356), reached its maximum in July (0.392), and subsequently declined from August (0.376) to October (0.215). Monthly NDVI anomalies relative to the long-term annual mean were positive from May to October and reached their maximum in July (**Figure 3a**). Based on the regional NDVI climatology and corresponding seasonal variations in temperature and precipitation, May–October was selected as a common analytical window for regional comparison. This window captures the main period of vegetation activity but does not imply identical phenological timing among vegetation types (**Figure 3b**).

The contrasts between July NDVI and April or October NDVI both exhibited significant increasing trends during 1999–2018 (p < 0.01; **Figure 3c**). The July–October NDVI contrast showed a stronger increase (0.037 decade^-1^) than the July–April contrast, indicating that changes in seasonal NDVI trajectories were not evenly distributed among months. For descriptive comparison, May–July and August–October were subsequently defined as the early-to-peak and late growing-season periods, respectively. These terms refer to analytical subdivisions of the May–October window rather than uniform physiological phenological stages.

Monthly NDVI trends were positive throughout the May–October analytical window, although their magnitudes and statistical significance differed among months (**Figure 3e**). Significant positive trends were detected in May (0.013 decade^-1^, p = 0.047), June (0.029 decade^-1^, p = 0.006), July (0.042 decade^-1^, p = 0.0003), August (0.029 decade^-1^, p = 0.0035), and September (0.024 decade^-1^, p = 0.0076). The October NDVI trend was positive but not statistically significant (0.005 decade^-1^, p = 0.096).

Because V_NDVI_ was calculated as the difference between NDVI in consecutive months, May V_NDVI_ represents the May–April NDVI difference. Mean V_NDVI_ was positive in May (0.110), June (0.099), and July (0.036), but negative in August (−0.016), September (−0.062), and October (−0.094). These values indicate that the regional NDVI generally increased from May to July and declined from August to October.

In contrast to the relatively consistent positive NDVI trends, monthly V_NDVI_ trends showed pronounced intra-seasonal asymmetry (**Figure 3d**). May V_NDVI_ exhibited a weak negative trend (−0.001 decade^-1^, p = 0.083), whereas significant positive trends occurred in June (0.016 decade^-1^, p = 0.036) and July (0.013 decade^-1^, p = 0.0047). V_NDVI_ trends were negative during August (−0.012 decade^-1^, p = 0.162), September (−0.006 decade^-1^, p = 0.092), and October (−0.019 decade^-1^, p = 0.0035), although only the October trend reached statistical significance.

Because V_NDVI_ represents the difference between two consecutive monthly NDVI values, its temporal trend reflects the difference between the NDVI trends of the target month and the preceding month. Therefore, the interpretation of V_NDVI_ trends depends on both the trend direction and the sign of mean V_NDVI_. The strongest positive V_NDVI_ trend occurred in June, reflecting a larger increase in June NDVI than in May NDVI over time, whereas the largest NDVI trend itself occurred in July. Similarly, the negative October V_NDVI_ trend occurred because the positive NDVI trend in October was smaller than that in September, resulting in a larger September-to-October NDVI difference.

#### 3.1.2 Spatial patterns of mean V_NDVI_ and temporal trends across the ARNC

**Figure 4:**
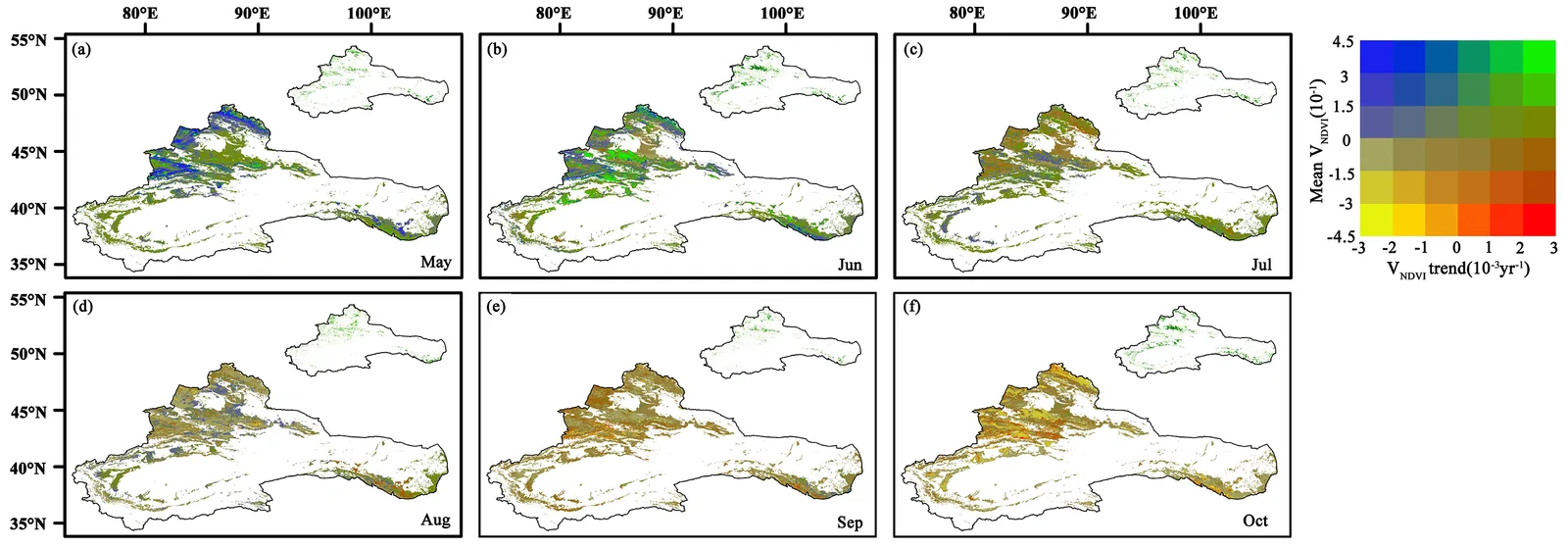
Bivariate spatial patterns of multi-year mean V_NDVI_ and its long-term trend during the May–October analytical window. **(a–f)** represent May, June, July, August, September, and October, respectively. The vertical axis of the bivariate legend represents multi-year mean V_NDVI_ during 1999–2018 in units of 10^-1^, and the horizontal axis represents the corresponding V_NDVI_ trend in units of 10^-3^ yr^-1^. Mean V_NDVI_ describes the average direction of month-to-month NDVI change, whereas the V_NDVI_ trend describes how that monthly difference changed over time. Green colors denote positive mean V_NDVI_ and positive trends; blue colors denote positive mean V_NDVI_ and negative trends; yellow-to-orange colors denote negative mean V_NDVI_ and negative trends; and red colors denote negative mean V_NDVI_ and positive trends. Positive and negative mean V_NDVI_ values indicate whether target-month NDVI was, on average, higher or lower than that of the preceding month and should not be interpreted directly as physiological processes.

Bivariate maps combining multi-year mean V_NDVI_ and its temporal trend revealed pronounced spatial and monthly heterogeneity across the May–October analytical window (**Figure 4**). Mean V_NDVI_ represents the average direction and magnitude of month-to-month NDVI change, whereas its temporal trend describes how this monthly difference changed during 1999–2018. These two metrics were therefore interpreted separately.

In May, mean V_NDVI_ was predominantly positive across vegetated areas, whereas V_NDVI_ trends showed substantial spatial variability (**Figure 4a**). Negative trends (approximately −0.002 to −0.001 yr^-1^) were mainly distributed in parts of the Altai Mountains and western Tianshan Mountains, while weak positive trends (0–0.001 yr^-1^) occurred across other vegetated regions. These spatial patterns corresponded to weaker April-to-May NDVI increases in some mountain areas (**Supplementary Figures 1a, g**).

In June, positive mean V_NDVI_ and positive temporal trends were widely distributed in the central Tianshan Mountains and along the northern margin of the Tarim Basin (**Figure 4b**). The strongest areas with both positive mean V_NDVI_ and positive trends were concentrated in parts of the central Tianshan Mountains, where mean V_NDVI_ ranged from approximately 0.30 to 0.45 and trends reached 0.002–0.003 yr^-1^. Similar but more spatially heterogeneous patterns were observed in July (**Figure 4c**), consistent with the significant regional increases in June and July V_NDVI_ (**Supplementary Figures 1b, h**, **2c, i**).

From August to October, mean V_NDVI_ was predominantly negative because NDVI values were generally lower than those of the preceding month after the seasonal maximum in July (**Figures 4d–f**). V_NDVI_ trends during August were spatially heterogeneous and generally weak. Negative trends became increasingly widespread in parts of the Tianshan and Altai Mountains during September and October, with October showing the clearest spatial pattern of increasingly negative V_NDVI_ trends. This pattern corresponded with the significant regional decrease in October V_NDVI_ trend.

### 3.2 Associations between V_NDVI_ and concurrent or antecedent climatic conditions in the ARNC

#### 3.2.1 Interannual associations between V_NDVI_ and selected concurrent or antecedent climatic conditions

**Figure 5:**
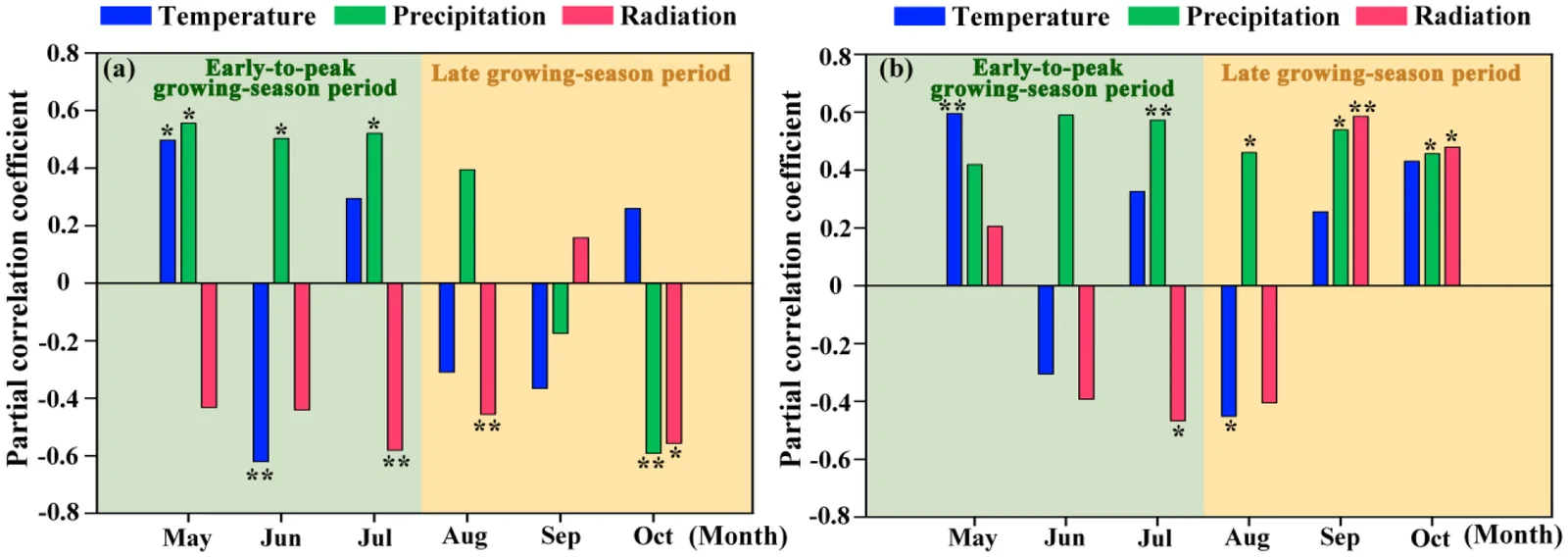
Partial correlations of monthly V_NDVI_ and NDVI with selected concurrent and antecedent climatic conditions during May–October. **(a)** Partial correlation coefficients between monthly V_NDVI_ and temperature, precipitation, and solar radiation. **(b)** Partial correlation coefficients between monthly NDVI and the corresponding climatic conditions. For each target month and climate variable, the concurrent month and the preceding three months were considered as candidate lags, and the lag with the largest absolute Pearson correlation coefficient was selected for subsequent partial correlation analysis. Green and yellow backgrounds denote the early-to-peak growing-season analytical period (May–July) and late growing-season analytical period (August–October), respectively; these periods represent common analytical subdivisions rather than uniform phenological stages across vegetation types. Positive and negative coefficients indicate positive and negative conditional associations, respectively, after controlling for the other two climatic variables. Asterisks indicate significance levels of p < 0.05 and p < 0.01.

Partial correlation analysis revealed seasonal differences in the associations between monthly V_NDVI_ and NDVI with selected concurrent or antecedent temperature, precipitation, and radiation conditions (**Figure 5**). For each target month and climatic variable, the lag with the largest absolute Pearson correlation coefficient among lag 0–3 was selected before partial correlation analysis (**Table 1**).

**Table 1:** Selected concurrent or antecedent climate lags corresponding to the strongest absolute Pearson correlations with monthly V_NDVI_ or NDVI across the study area.

Month	Selected climate lag for V_NDVI_			Selected climate lag for NDVI		
	Temperature (months)	Precipitation (months)	Radiation (months)	Temperature (months)	Precipitation (months)	Radiation (months)
May	3	1	1	1	1	3
June	1	3	3	1	2	2
July	0	1	1	0	1	1
August	1	1	1	3	1	0
September	2	3	2	0	2	0
October	0	2	1	0	3	0

Lag 0 denotes climatic conditions in the target month (concurrent climate), whereas lags 1–3 denote conditions one to three months before the target month.

During the early-to-peak growing season (May–July), precipitation generally showed positive associations with V_NDVI_, whereas radiation exhibited predominantly negative associations (**Figure 5a**). May V_NDVI_ was positively associated with selected temperature and precipitation conditions (p < 0.05). In June, V_NDVI_ showed a negative association with temperature (p < 0.01) and a positive association with precipitation (p < 0.05). July V_NDVI_ was positively associated with June precipitation (p < 0.05) and negatively associated with June radiation (p < 0.01). Similar patterns were observed for July NDVI, which showed positive associations with June precipitation (p < 0.01) and negative associations with June radiation (p < 0.05; **Figure 5b**).

During the late growing season (August–October), climatic associations became more heterogeneous. August V_NDVI_ was negatively associated with antecedent radiation (p < 0.01), whereas August NDVI showed negative associations with antecedent temperature and positive associations with July precipitation (p < 0.05). No significant climatic associations were detected for September V_NDVI_, although September NDVI showed positive associations with July precipitation and concurrent radiation. In October, V_NDVI_ showed negative associations with August precipitation and September radiation (p < 0.01), whereas October NDVI showed positive associations with concurrent temperature, radiation, and July precipitation (p < 0.05).

Overall, NDVI and V_NDVI_ exhibited contrasting climatic associations in several months, particularly during the late growing season. These differences indicate that climatic conditions associated with vegetation greenness levels were not always consistent with those associated with month-to-month NDVI changes.

#### 3.2.2 Spatial heterogeneity in V_NDVI_–climate associations

**Figure 6:**
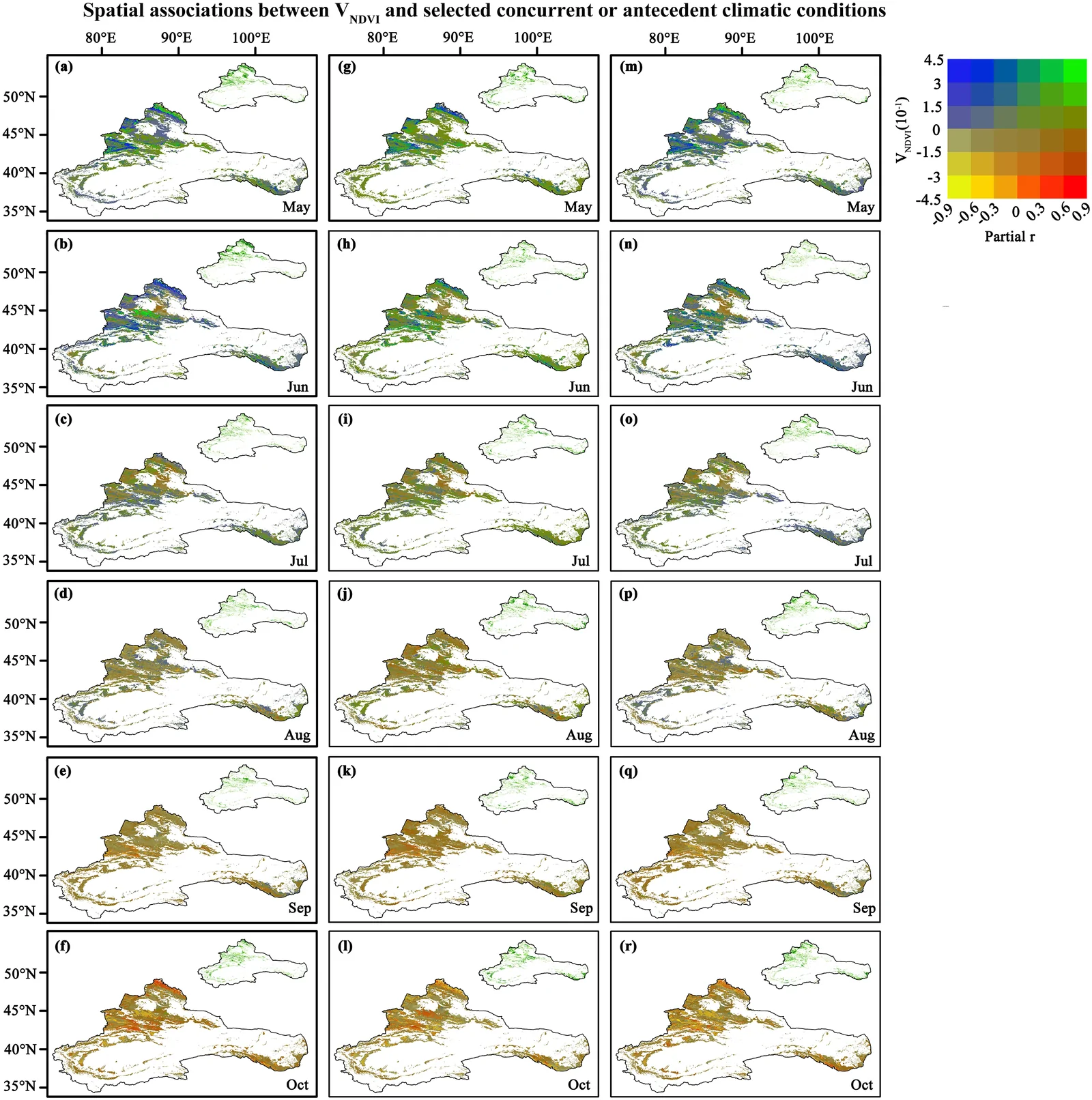
Spatial patterns of partial correlations between monthly V_NDVI_ and selected concurrent or antecedent climatic conditions across the ARNC. The six rows represent May–October, respectively, and the three columns represent temperature (left column), precipitation (middle column), and solar radiation (right column). For each panel, the bivariate legend combines the multi-year mean V_NDVI_ during 1999–2018 (y-axis) with the partial correlation coefficient (partial r) between V_NDVI_ and the selected climatic condition (x-axis). The same bivariate legend applies to all panels. Green sectors (V_NDVI_ > 0, r > 0) indicate areas with positive mean V_NDVI_ and positive partial correlations; blue sectors (V_NDVI_ > 0, r < 0) indicate areas with positive mean V_NDVI_ and negative partial correlations; yellow sectors (V_NDVI_ < 0, r < 0) indicate areas with negative mean V_NDVI_ and negative partial correlations; and red sectors (V_NDVI_ < 0, r > 0) indicate areas with negative mean V_NDVI_ and positive partial correlations. The inset maps in the upper-right corner of each panel show the spatial distribution of pixels with statistically significant partial correlations (p < 0.05).

Spatial patterns of partial correlations showed substantial monthly and regional differences in the associations between V_NDVI_ and selected concurrent or antecedent climatic conditions across the ARNC (**Figure 6**).

During the early-to-peak growing season, temperature and precipitation exhibited contrasting spatial associations with May V_NDVI_. Positive associations with temperature and precipitation were widely distributed across vegetated regions, whereas some areas showed opposite patterns. In the Altai Mountains, positive associations between antecedent temperature and May V_NDVI_ coexisted with negative associations between antecedent precipitation and May V_NDVI_ (**Supplementary Figures 2**–**4**). These spatial patterns corresponded with the heterogeneous May V_NDVI_ trends observed across the region.

For June and July, V_NDVI_ showed predominantly negative associations with temperature and positive associations with precipitation in several regions, although the spatial patterns varied substantially (**Figures 6b, h**). Positive V_NDVI_ trends in the central Tianshan Mountains and northern margin of the Tarim Basin overlapped with areas showing positive associations between V_NDVI_ and selected climatic conditions. In the northern margin of the Tarim Basin, precipitation showed relatively strong positive associations with V_NDVI_ during June and July (**Supplementary Figure 3**).

During August–October, spatial patterns of V_NDVI_–climate associations became more variable. Positive associations between precipitation and August V_NDVI_ occurred across many regions, whereas temperature showed contrasting patterns. As the season progressed, spatial differences in the sign and magnitude of climatic associations became increasingly apparent, particularly in the Tianshan and Altai Mountains (**Supplementary Figures 3**, **4**).

Overall, the spatial patterns indicate substantial heterogeneity in the climatic conditions associated with monthly V_NDVI_ variation across the ARNC.

### 3.3 Relative independent explanatory contributions of climatic conditions to NDVI variability across vegetation types

Hierarchical partitioning was used to quantify the relative independent explanatory contributions of selected concurrent and antecedent climatic variables to interannual NDVI variability across vegetation types (**Figure 2**). Temperature, precipitation, and radiation were evaluated for each target month using the climate lags selected at the pixel level, and the resulting independent contributions were summarized for the entire study region and for individual vegetation types. The corresponding model R² values were used to characterize the overall proportion of interannual NDVI variability explained jointly by the three climatic predictors.

At the regional scale, the relative independent contributions of the climatic variables varied substantially among months (**Figure 2a**). Temperature accounted for the largest share of the summed independent contributions in May and October, reaching approximately 60%, whereas the variable with the largest contribution differed among the intervening months. The regional model had an R² of 0.78, indicating that the three selected climatic variables jointly accounted for a substantial proportion of the modeled interannual NDVI variability.

Grasslands showed relatively high contributions from temperature during the early-to-peak growing-season period (**Figure 2b**). In June and July, temperature represented more than 60% of the summed independent contributions of the three climatic predictors. The grassland model had the highest R² among the vegetation types (R² = 0.83), suggesting that interannual grassland NDVI variability was comparatively well characterized by the selected temperature, precipitation, and radiation variables.

Shrublands exhibited marked seasonal variation in the relative contributions of the climatic predictors (**Figure 2c**). Temperature made a relatively large contribution in several months, whereas precipitation and radiation became more important in others. The shrubland model yielded an R² of 0.64, indicating moderate explanatory capacity and suggesting that additional environmental factors may also contribute to shrubland NDVI variability.

Croplands displayed a distinct early-season pattern (**Figure 2d**). May temperature represented approximately 72% of the summed independent contributions of the three climatic predictors to May NDVI variability. However, the cropland model had a comparatively lower R² of 0.57. Thus, the large relative contribution of temperature should be interpreted as its importance relative to precipitation and radiation within the fitted model, rather than as evidence that temperature explained 72% of the total variation in cropland NDVI. The lower model fit also suggests that irrigation, crop type, sowing dates, and other management practices may account for a substantial portion of the unexplained variability.

Forests showed contributions from multiple climatic variables, with temperature and precipitation generally accounting for substantial but seasonally varying shares (**Figure 2e**). The forest model had an R² of 0.69, indicating moderate-to-high explanatory capacity. Sparse vegetation exhibited more dispersed and generally smaller relative contributions among the climatic variables across months (**Figure 2f**), and its model had the lowest R² among the analyzed classes (R² = 0.54). This comparatively low explanatory capacity may reflect the influence of non-climatic factors, weak vegetation signals, background reflectance, or local water sources not represented by the selected climatic variables.

Overall, both the relative climatic contributions and model explanatory capacity differed among vegetation types. The higher R² values for grasslands and forests indicate that their interannual NDVI variability was more strongly captured by the selected climatic predictors, whereas the lower R² values for croplands and sparse vegetation point to a greater potential role of management, local hydrological conditions, surface-background effects, or other omitted factors. These results highlight substantial heterogeneity in vegetation–climate associations across the ARNC.

## 4 Discussion

Long-term increases in annual or growing-season mean NDVI have been widely reported across China, including its arid and semi-arid regions (Ren et al., 2025; Wei et al., 2025). However, seasonal mean metrics may conceal differences in how vegetation greenness changes are distributed among individual months. In the ARNC, NDVI increased during most months of the May–October analytical window, whereas the trends in month-to-month NDVI differences were positive in June and July but negative in October. Because the trend in V_NDVI_ is mathematically determined by the difference between the NDVI trends of two consecutive months, these results indicate a temporal redistribution of seasonal greenness changes rather than a uniform acceleration or weakening of vegetation growth. This distinction is important for interpreting regional greening in water-limited ecosystems, where similar increases in seasonal mean NDVI may arise from substantially different changes in the seasonal NDVI trajectory.

### 4.1 Temporal asymmetry in month-to-month vegetation greenness changes

The early-to-peak growing-season period showed the clearest positive changes in the month-to-month NDVI trajectory. Although monthly NDVI increased throughout May–July, the increase was substantially larger in June and July than in the preceding months, producing significant positive V_NDVI_ trends in these two months. Thus, the positive June and July V_NDVI_ trends do not necessarily indicate an increasing physiological growth rate; rather, they indicate that the long-term increases in June and July NDVI exceeded those in May and June, respectively.

The partial-correlation results provide additional information on the climatic conditions associated with interannual variability in these monthly NDVI differences. Precipitation was positively associated with V_NDVI_ in May, June, and July, although the selected concurrent or antecedent month differed among target months. In particular, June and July V_NDVI_ were positively associated with antecedent precipitation. These relationships are consistent with the general importance of water availability for vegetation greenness in drylands (Jiao et al., 2021), but they should be interpreted as conditional statistical associations rather than evidence that antecedent precipitation directly caused the observed long-term V_NDVI_ trends.

Temperature and radiation showed different associations from precipitation during the early-to-peak growing-season period. May V_NDVI_ was positively associated with antecedent temperature, whereas June V_NDVI_ was negatively associated with selected antecedent temperature. Radiation was negatively associated with V_NDVI_ in several early-season months, including a significant negative association between July V_NDVI_ and June radiation. These month-specific changes in association suggest that the climatic context related to increasing greenness early in the season may differ from that associated with greenness changes near the seasonal peak.

In water-limited environments, warm and high-radiation conditions can be associated with both favorable and unfavorable vegetation conditions. Moderate warming may coincide with earlier snowmelt, soil thaw, or improved thermal conditions, whereas high temperature and radiation may also increase evaporative demand when water supply is limited. The latter interpretation is compatible with previous evidence of increasing atmospheric water constraints on vegetation activity (Yuan et al., 2019; Jiao et al., 2021). However, because VPD, soil moisture, evapotranspiration, and plant physiological responses were not directly examined in this study, these processes remain plausible explanations rather than mechanisms demonstrated by the present analyses.

The late growing-season period showed a different form of asymmetry. Mean V_NDVI_ was negative from August to October because regional NDVI had already reached its seasonal maximum in July and subsequently declined. Nevertheless, NDVI continued to exhibit positive long-term trends in August and September, indicating that late-season greenness increased over the study period even though NDVI was declining within an average year.

The significant negative October V_NDVI_ trend resulted from the difference between the September and October NDVI trends. September NDVI increased significantly, whereas the positive October NDVI trend was smaller and not statistically significant. Consequently, the September-to-October decline in NDVI became larger over time. This result should therefore be interpreted as an increasing contrast between September and October greenness, rather than as direct evidence of accelerated physiological senescence.

Antecedent precipitation was positively associated with late-season NDVI in several months. August NDVI was positively associated with July precipitation, while September and October NDVI were also positively associated with selected antecedent precipitation. Concurrent or antecedent radiation showed positive associations with September and October NDVI, although August NDVI was negatively associated with antecedent radiation. These contrasting relationships indicate that climatic associations with absolute late-season greenness varied among months and were not necessarily equivalent to those associated with V_NDVI_.

Several processes could potentially contribute to the contrasting September and October patterns. Previous studies have shown that antecedent precipitation can influence subsequent vegetation activity through delayed changes in water availability, and that high atmospheric demand can constrain vegetation activity in drylands (Li et al., 2024; Qiu et al., 2026). These processes provide plausible ecological contexts for the positive associations between antecedent precipitation and late-season NDVI and for the weaker NDVI increase in October than in September.

Nevertheless, the present results do not directly demonstrate soil-moisture memory, atmospheric-drought effects, accelerated leaf senescence, or hydraulic-safety regulation (Yan et al., 2025). The selected climate lags identify the months with the strongest statistical associations within the tested lag range, but they cannot distinguish delayed ecological responses from temporal covariance among climate variables and vegetation greenness. Direct observations of soil moisture, VPD, plant water status, and phenological transitions would be required to test these alternative explanations.

### 4.2 Spatial heterogeneity in month-to-month NDVI changes

The spatial analysis showed that the weak negative regional trend in May V_NDVI_ was not spatially uniform. Negative trends were concentrated mainly in parts of the Altai Mountains and western Tianshan Mountains, whereas weak positive trends occurred across many other vegetated areas. Because May V_NDVI_ is the difference between May and April NDVI, these negative trends indicate that the April-to-May NDVI increase became smaller in these mountain areas over time.

In parts of the Altai Mountains, May V_NDVI_ was positively associated with antecedent temperature but negatively associated with antecedent precipitation. One possible interpretation is that temperature limitation remains relevant to early-season greenness at high latitudes or elevations, where snow cover, soil thaw, and low temperatures may delay vegetation activity (Wang and Chen, 2024; Li et al., 2025). Under such conditions, warmer antecedent months may coincide with conditions favorable for an earlier increase in greenness. The negative precipitation association is more difficult to interpret and may reflect precipitation phase, snow accumulation, cloud–radiation covariance, or other unmeasured environmental conditions (Tierney et al., 2001; Wang and Chen, 2024; Li et al., 2025). Because snow cover, soil temperature, precipitation phase, and freeze–thaw processes were not analyzed, the present data cannot determine which of these explanations is responsible for the observed spatial pattern.

Positive June and July V_NDVI_ trends were particularly evident in the central Tianshan Mountains and along the northern margin of the Tarim Basin. In these areas, the spatial distribution of positive V_NDVI_ trends partly overlapped with positive associations between V_NDVI_ and selected antecedent precipitation. This spatial correspondence suggests that antecedent water availability may be relevant to the interannual variability of early-summer month-to-month greenness changes, although the trend and correlation analyses address different components of variability and should not be interpreted as direct evidence of climatic causation.

In the central Tianshan Mountains, spring temperature and precipitation may be associated with several processes relevant to subsequent vegetation greenness, including snowmelt timing and seasonal water availability (Tierney et al., 2001; Mtupili et al., 2025). Along the northern margin of the Tarim Basin, positive associations with antecedent precipitation are consistent with strong water limitation in oasis margins and desert–oasis ecotones (Zhang et al., 2026). However, the present analysis does not distinguish direct rainfall inputs from runoff, irrigation, groundwater access, or meltwater contributions. The ecological interpretation of these spatial associations therefore remains conditional on local hydrological settings.

From August to October, the spatial pattern of V_NDVI_ shifted from heterogeneous and generally weak trends in August to more widespread negative trends in September and October, particularly in parts of the Tianshan and Altai Mountains. Because mean V_NDVI_ was already negative during these months, increasingly negative trends indicate that the decline in NDVI from the preceding month became larger over time in these areas.

Climatic associations also became more spatially variable during the late growing season. Positive associations with precipitation occurred in many areas in August, whereas the signs and magnitudes of temperature and radiation associations differed among regions and months. By September and October, the spatial patterns were increasingly heterogeneous, especially in mountain regions. Seasonal cooling, frost occurrence, declining radiation, antecedent water availability, and local topographic conditions could all contribute to these late-season differences (Ji et al., 2026). However, the monthly climate variables used here cannot identify the timing of frost events or distinguish physiological senescence from other causes of declining NDVI. Accordingly, the increasingly negative late-season V_NDVI_ trends are best interpreted as changes in the seasonal greenness trajectory rather than direct evidence of earlier or faster senescence.

### 4.3 Differences in climatic associations among vegetation types

Hierarchical partitioning revealed substantial differences among vegetation types in both the relative contributions of the selected climatic variables and the overall explanatory capacity of the fitted models. The relative contribution values describe the importance of temperature, precipitation, and radiation compared with one another within each model, whereas the corresponding R² values indicate how well these climatic predictors jointly characterized interannual NDVI variability. These two quantities therefore provide complementary but distinct information.

Grasslands showed the strongest overall climate–NDVI relationship among the vegetation types, with a model R² of 0.83. Temperature accounted for a relatively large share of the summed independent contributions in June and July, exceeding 60% in both months. This pattern indicates that early-to-peak growing-season grassland NDVI variability was more strongly associated with temperature than with precipitation or radiation within the fitted models. However, the large relative contribution of temperature does not by itself identify a specific physiological pathway. Temperature may represent direct thermal constraints but may also covary with snowmelt timing, soil thaw, evaporative demand, or the timing of seasonal vegetation activity.

Shrublands had a lower model R² of 0.64 and showed greater seasonal variation in the relative contributions of the three climatic predictors. Temperature contributed substantially in several months, whereas precipitation and radiation became relatively more important in others. This seasonal variation suggests that shrubland greenness may be associated with multiple climatic constraints that change across the growing season. Although such patterns may be compatible with differences in water-access strategies or delayed responses to antecedent conditions (Huo et al., 2025; Pan et al., 2025), the present analysis does not directly demonstrate deep-root water uptake, soil-moisture memory, or other belowground mechanisms.

Croplands exhibited a distinct pattern, with May temperature accounting for approximately 72% of the summed independent contributions of the three climatic predictors. This percentage indicates that temperature was the most important of the three included climate variables for May cropland NDVI; it does not mean that temperature explained 72% of the total variation in cropland NDVI. The cropland model had a comparatively lower R² of 0.57, indicating that a substantial portion of interannual NDVI variability was not characterized by the selected climatic predictors. Irrigation, sowing dates, crop composition, fertilization, harvesting practices, and other management factors may contribute to this unexplained variation. The cropland results should therefore be interpreted as climate–greenness associations within managed agricultural systems rather than as evidence that temperature alone controls early-season crop development.

Forests showed an intermediate-to-high model R² of 0.69, with temperature and precipitation generally making substantial but seasonally varying contributions. This result indicates that forest NDVI variability was moderately well characterized by the selected climatic variables, although the relative importance of individual predictors changed among months. Such variation may reflect differences in seasonal thermal and water constraints, but the current data do not resolve the underlying physiological responses.

Sparse vegetation had the lowest model R² among the analyzed vegetation types (R² = 0.54). The relative climatic contributions were more dispersed across variables and months, suggesting that NDVI variability in sparsely vegetated areas was less consistently associated with the selected temperature, precipitation, and radiation predictors. The lower explanatory capacity may reflect the influence of local hydrological conditions, groundwater access, episodic runoff, short-duration precipitation pulses, or stronger soil-background effects on NDVI (Yan et al., 2025). These possibilities remain hypotheses because they were not directly evaluated in the present study.

Overall, the higher R² values for grasslands and forests indicate that their interannual NDVI variability was more strongly captured by the selected climatic predictors, whereas the lower R² values for croplands and sparse vegetation suggest a greater potential influence of management, local hydrology, surface-background effects, or other omitted variables. Thus, vegetation types differed not only in which climatic variable had the largest relative contribution, but also in the extent to which temperature, precipitation, and radiation jointly explained their interannual greenness variability.

## 5 Conclusions

Regional greening across arid Northwest China was not expressed uniformly throughout the growing season but instead reflected a marked redistribution of month-to-month vegetation greenness changes. From 1999 to 2018, V_NDVI_ increased significantly in June and July but declined in October, indicating that long-term NDVI gains were concentrated more strongly around the early-to-peak growing season than in late autumn. These temporal shifts were spatially heterogeneous, with contrasting patterns among the Altai Mountains, Tianshan Mountains, and the northern margin of the Tarim Basin.

Climatic associations also varied by month, region, and vegetation type. Precipitation was generally positively associated with early-season greenness changes, whereas temperature and radiation showed seasonally contrasting relationships. Hierarchical partitioning further revealed that both the relative importance and joint explanatory capacity of climatic variables differed among vegetation types, with stronger climate–NDVI coupling in grasslands and forests than in croplands and sparse vegetation.

Together, these findings demonstrate that seasonal mean greening can conceal substantial reorganization of the intra-seasonal greenness trajectory. Resolving month-to-month changes therefore provides a more temporally explicit framework for assessing vegetation–climate relationships in water-limited ecosystems.

## 6 Limitations and future directions

This study provides a regional-scale assessment of intra-seasonal vegetation greenness changes and their climatic associations across arid Northwest China. Nevertheless, several aspects could be further strengthened in future research. NDVI primarily reflects satellite-observed surface greenness, and V_NDVI_ characterizes month-to-month changes along the seasonal NDVI trajectory. These metrics are well suited for identifying broad temporal and spatial patterns, although they do not directly represent physiological growth, biomass accumulation, canopy structural development, or senescence.

The monthly temporal resolution enabled consistent regional comparisons over two decades but may not fully capture short-lived climatic events or abrupt phenological transitions. Similarly, the use of a common May–October analytical window facilitated comparisons among regions and vegetation types, although local phenological timing may vary with elevation, vegetation type, and land management. The selected concurrent and antecedent climate lags identify the periods showing the strongest statistical associations with NDVI or V_NDVI_, but additional observations are required to determine whether these associations reflect delayed ecological responses or other forms of temporal covariance.

Future research could integrate higher-frequency remote sensing, solar-induced chlorophyll fluorescence, soil-moisture observations, atmospheric-demand indicators, and field phenological measurements to further evaluate the ecological processes underlying the observed patterns. Root-zone monitoring and stable-isotope approaches may also help assess plant water-use strategies, while the inclusion of irrigation, crop management, groundwater, and microtopographic information would improve the separation of climatic, hydrological, and human influences on seasonal vegetation greenness dynamics.

## Data Availability

Publicly available datasets were analyzed in this study. This data can be found here: NDVI data: http://www.resdc.cn/data.aspx?DATAID=254. Meteorological data: https://www.chelsa-climate.org/datasets/chelsa_monthly.
