# Supplementary material for: Intra-seasonal asymmetry in satellite-observed vegetation greenness changes and their climatic associations in arid Northwest China
Source: Front Plant Sci. 2026 Aug 13;17:1897977. doi: 10.3389/fpls.2026.1897977 (PMC13518329; doi:10.3389/fpls.2026.1897977)
Supplement: Supplementary file 1 [file Table1.docx]

**Supplementary Figure 1.** Spatial patterns of monthly NDVI trends and their statistical significance during the May–October analytical window (1999–2018). Panels (a–f) show the spatial distribution of NDVI trend slopes for each month from May to October, whereas the corresponding panels in the right column show the significance levels of the detected trends based on the Mann–Kendall test. Trend values represent long-term changes in satellite-observed vegetation greenness.

**Supplementary Figure 2.** Spatial patterns of selected antecedent climatic lags for monthly V_NDVI_ associated with temperature, precipitation, and radiation. For each target month, climatic conditions from the same month and the preceding three months were evaluated (lag 0–3). The selected lag represents the month showing the strongest statistical association between the corresponding climatic variable and monthly V_NDVI_ at each pixel.The same legend applies to all panels.

**Supplementary Figure 3.** Spatial patterns of the maximum correlations between NDVI and climatic factors during May–October. The first, second, and third columns show the spatial distributions of the maximum correlation coefficients between NDVI and temperature, precipitation, and radiation, respectively. From top to bottom, rows represent correlations between NDVI and climatic factors in the current month and with 1-, 2-, and 3-month lags. Insets in the upper-right corner of each panel show the spatial distribution of significant correlations (p < 0.05).

**Supplementary Figure 4.** Spatial patterns of selected concurrent and antecedent climatic lags associated with monthly NDVI. Climatic conditions from lag 0 to lag 3 were evaluated for each target month, and the lag with the strongest statistical association between climate variables and monthly NDVI was selected independently for each pixel.The same legend applies to all panels.
